# Inequities in access to HIV prevention services for transgender men: results of a global survey of men who have sex with men

**DOI:** 10.7448/IAS.19.3.20779

**Published:** 2016-07-17

**Authors:** Ayden I Scheim, Glenn-Milo Santos, Sonya Arreola, Keletso Makofane, Tri D Do, Patrick Hebert, Matthew Thomann, George Ayala

**Affiliations:** 1 Department of Epidemiology and Biostatistics, The University of Western Ontario, London, Canada; 2 Community Health Systems, School of Nursing (G-M Santos), Department of Medicine (TD Do), University of California, San Francisco, CA, USA; 3 San Francisco Department of Public Health, San Francisco, CA, USA; 4 The Global Forum on MSM & HIV (MSMGF), Oakland, CA, USA; 5 Human Sexuality Program, California Institute of Integral Studies, San Francisco, CA, USA; 6 Department of Sociomedical Sciences, Mailman School of Public Health, Columbia University, New York, NY, USA

**Keywords:** HIV prevention, health services, transgender, men who have sex with men, HIV testing

## Abstract

**Introduction:**

Free or low-cost HIV testing, condoms, and lubricants are foundational HIV prevention strategies, yet are often inaccessible for men who have sex with men (MSM). In the global context of stigma and poor healthcare access, transgender (trans) MSM may face additional barriers to HIV prevention services. Drawing on data from a global survey of MSM, we aimed to describe perceived access to prevention services among trans MSM, examine associations between stigma and access, and compare access between trans MSM and cisgender (non-transgender) MSM.

**Methods:**

The 2014 Global Men's Health and Rights online survey was open to MSM (inclusive of trans MSM) from any country and available in seven languages. Baseline data (*n=*3857) were collected from July to October 2014. Among trans MSM, correlations were calculated between perceived service accessibility and anti-transgender violence, healthcare provider stigma, and discrimination. Using a nested matched-pair study design, trans MSM were matched 4:1 to cisgender MSM on age group, region, and HIV status, and conditional logistic regression models compared perceived access to prevention services by transgender status.

**Results:**

About 3.4% of respondents were trans men, of whom 69 were included in the present analysis. The average trans MSM participant was 26 to 35 years old (56.5%); lived in western Europe, North America, or Oceania (75.4%); and reported being HIV-negative (98.6%). HIV testing, condoms, and lubricants were accessible for 43.5, 53.6, and 26.1% of trans MSM, respectively. Ever having been arrested or convicted due to being trans and higher exposure to healthcare provider stigma in the past six months were associated with less access to some prevention services. Compared to matched cisgender controls, trans MSM reported significantly lower odds of perceived access to HIV testing (OR=0.57, 95% CI=0.33, 0.98) and condom-compatible lubricants (OR=0.54, 95% CI=0.30, 0.98).

**Conclusions:**

This first look at access to HIV prevention services for trans MSM globally found that most reported inadequate access to basic prevention services and that they were less likely than cisgender MSM to have access to HIV testing and lubricants. Results indicate the need to enhance access to basic HIV prevention services for trans MSM, including MSM-specific services.

## Introduction

In Europe and North America, a majority of transgender (trans) men report attraction to other men [1–3], and a substantial proportion report recent sexual contact with cisgender (non-trans) men. For example, in Ontario, Canada, 63% of trans men identified as gay, bisexual, or queer, and/or had a male sex partner in the past year, while 21% had a cisgender male sex partner in the past year [1]. Trans men who have sex with men (trans MSM) have been identified as a key population facing stigma, and potentially increased vulnerability to HIV, across settings [4]. Yet, research evidence regarding HIV prevalence, risk, and prevention service access is scant and almost entirely limited to small convenience samples in Canada and the United States [5,6]. A recent international review identified 10 studies with laboratory-confirmed HIV seroprevalence data for trans men, which ranged from 0 to 4% [7]. Of these studies, only the two smallest (both *n*=14) were specific to trans MSM [8,9], who are at increased biological risk for HIV and may face structural barriers rendering them more vulnerable than other trans men. Self-reported prevalence among trans MSM has ranged from 0 to 5.9% [10–13]. While these prevalence estimates are low when compared to cisgender MSM and transgender women [6,14], the higher estimates are many-fold greater than HIV prevalence in the broader adult populations of Canada and the United States. Moreover, trans MSM are increasingly integrated in MSM sexual networks in some settings [12,15], and this may potentiate increased HIV risk, particularly if access to basic HIV prevention services is limited.

Trans people face systemic barriers to healthcare in systems that, by and large, operate with the taken-for-granted assumption that patients or clients will be cisgender [16]. The results of this pervasive assumption have been described as informational and institutional “erasure” [16], leading to limited medical education on trans health issues [17], absence of policies to accommodate trans patients (e.g., regarding access for individuals without identification that matches their gender presentation [18]), discomfort and uncertainty in healthcare encounters [19,20] and outright mistreatment or denial of care [21,22]. Specific to sexual health services, trans men have reported experiencing limited provider knowledge, stigmatizing attitudes, and invalidation of their gender identities, which limit their ability to disclose their sexual health needs (e.g., related to having vaginal sex with cisgender men) and access HIV/STI testing or other prevention services [10,23]. Indeed, some evidence points to low uptake of HIV testing among trans MSM, even in a setting with universal health coverage where HIV testing is relatively accessible for cisgender MSM [1]. Discrimination in healthcare is situated in a context of widespread stigma across a range of settings [2,24], which may further reduce perceived accessibility of health services, or willingness to access them (for fear of encountering discrimination). Trans MSM may additionally contend with stigma related to their sexual orientation or behaviour. For cisgender MSM, sexual stigma has been associated with reduced access to HIV testing and treatment, condoms, and lubricants [25].

Drawing on data from a global survey of MSM (inclusive of trans MSM), we aimed to describe perceived accessibility of prevention services among trans MSM, examine associations between perceived stigma and access, and compare access between trans MSM and matched cisgender MSM. We hypothesized (1) that greater exposure to anti-transgender violence, discrimination, and healthcare provider stigma would be associated with reduced access to HIV testing, condoms, and lubricants for trans MSM and (2) that trans men would report less access to these services than demographically comparable cisgender men.

## Methods

### Data source

The 2014 Global Men's Health and Rights Survey was a web-based longitudinal survey of MSM recruited through online convenience and purposive sampling (e.g., via organizational networks, email listservs, MSM websites). Eligible participants needed to identify as male or as trans men, not report sexual attraction exclusively to women, be 18 years of age or older, and be able to complete the survey in Arabic, Chinese, English, French, Portuguese, Russian, or Spanish. No geographical restrictions were applied, and the inclusion of trans men was specified in recruitment materials. At baseline, participants completed a 30-minute survey including items about demographics, stigma, and access to healthcare (including but not limited to HIV prevention, treatment, and support). Six- and 12-month follow-up data were collected from a subset who consented to participate in a longitudinal component, but only baseline data are included in the present analysis. Ethical approval was obtained from the Western Institutional Review Board.

### Measures

#### Demographics

Trans respondents were identified using a two-step method [26]; current gender identity and assigned sex at birth were ascertained. Those who reported a female natal sex and identified either as male or as trans men (parenthetically defined for respondents as female-to-male trans people) were classified as trans MSM. Age, country of residence, gender of primary or main partner in the past six months, gender(s) to which participants were attracted, and most recent HIV test result were self-reported.

#### Stigma

Items pertaining to violence, discrimination, and healthcare provider stigma related to being trans were adapted from parallel MSM-specific items (trans men received both sets). Ever experiencing violence attributed to anti-trans stigma was assessed with a four-item scale (Cronbach's *a=*0.74) with items relating to physical violence, sexual assault, threats or blackmail, and malicious disclosure of trans status (“Has the fact that you are a trans man ever been disclosed against your will by someone who intended to cause you harm?”). Five items (*a=*0.86) assessed stigma and discrimination from healthcare providers in the past six months, including whether a provider had “treated you poorly …,” “refused to treat you …,” “judged you …,” “reprimanded (lectured/scolded) you … because you are a trans man,” or “disclosed that you are a trans man to others with your permission.” For violence and provider stigma scales, participants indicated the frequency with which they experienced each type of violence or stigma (from never to more than five times). Responses were summed and divided by the number of items to generate a mean frequency score ranging from 0 (never) to 4 (all types experienced more than five times). Three items measured trans-related discrimination (“Have you ever been …arrested or convicted/denied employment/harassed by police because you are a trans man”) and were not summed to form a scale as they represent discrete events and have poor internal consistency (*a=*0.49). They were dichotomized for analyses to indicate ever versus never having each discrimination experience.

#### Access to HIV prevention services

Participants were asked “In your community, how accessible is free or affordable HIV testing?” Parallel items assessed perceived accessibility of condoms and condom-compatible lubricants. Response options were on a 5-point scale from “completely inaccessible” to “completely accessible,” and were dichotomized as completely accessible versus somewhat accessible or less, consistent with prior analyses [25,27].

### Statistical analysis

All analyses were conducted in STATA version 13.1 [28]. Descriptive statistics were calculated for GMHR participants who were identified as trans MSM. Violence and healthcare provider stigma scale scores were skewed, and therefore, medians are reported and non-parametric statistics were employed to examine associations. The Wilcoxon rank-sum test was used to examine associations between transphobic violence and provider stigma scores and each HIV service access outcome (testing, condoms, and lubricants), while Fisher's exact test was used for the binary trans discrimination variables (arrest or conviction, police harassment, employment discrimination). Statistical significance was set at *p*<0.05.

In the nested matched study, trans MSM were randomly matched 4:1 to cisgender MSM controls on age group (25 and under, 26–35, 36–45, 46+), region (eastern Europe and Central Asia; Latin America; Sub-Saharan Africa; western Europe, North America, or Oceania; and other), and self-reported HIV status (negative or unknown vs. positive). Each trans and cisgender MSM matched group was assigned a match ID number that was used to define strata. Conditional logistic regression models were fit to estimate the odds of perceived access to HIV testing, condoms, and condom-compatible lubricants, comparing trans MSM to matched cisgender MSM. The nested pair-matched study design has been shown to be an effective method to evaluate disparities between transgender participants and a subset of cisgender controls [29]. Sensitivity analyses were conducted by adjusting conditional logistic regression models for an indicator of socio-economic status (SES; ability to meet basic needs with current income, e.g., food, shelter, transportation, healthcare, and education). SES was not selected as a matching variable due to its potential to mediate the association between transgender status and service access.

## Results

Of 3857 survey respondents, 133 (3.4%) were identified as trans MSM, including those who reported their gender identity as trans man (*n*=81), and others who indicated male gender identity in combination with female natal sex (*n*=52). Participants who indicated being assigned male at birth but identified as trans men (*n*=15) could not be appropriately categorized and were excluded from further analysis. Trans MSM who completed all items related to anti-transgender stigma and discrimination (*n*=69) were included in subsequent analyses. Demographic characteristics of trans MSM and matched cisgender MSM, and experiences of stigma among trans MSM, are described in Table 1. Most trans MSM were between the ages of 18 and 25 (23.2%) or 26 and 35 (56.5%) and lived in western Europe, North America, or Oceania (75.4%). One reported being HIV positive (1.4%). Of the 98.6% who did not identify as HIV positive, 91.1% reported that their last HIV test result was negative, while 8.8% had never been tested. Most trans MSM (69.7%, *n*=48) reported a cisgender male primary or main sexual partner in the past six months. All (100%) reported attraction to cisgender men, and 70% were primarily or exclusively attracted to cisgender men.

**Table 1:** Demographic characteristics and experiences of stigma among transgender (*n*=69) and pair-matched cisgender (*n*=276) men in a global survey of men who have sex with men

	Trans men % (*n*) or Median (IQR)	Cisgender men % (*n*)
Age group (%)		
18 to 25 years	23.2 (16)	23.2 (64)
26 to 35 years	56.5 (39)	56.5 (156)
36 to 45 years	11.6 (8)	11.6 (32)
46 years+	8.7 (6)	8.7 (24)
Region (%)		
Eastern Europe and Central Asia	4.3 (3)	4.3 (12)
Latin America and Caribbean	4.3 (3)	4.3 (12)
Sub-Saharan Africa	2.9 (2)	2.9 (8)
Western Europe, North America, Oceania	75.4 (52)	75.4 (208)
Other	13.0 (9)	13.0 (36)
Self-reported HIV status (%)		
Negative or unknown	98.6 (68)	98.6 (272)
Positive	1.5 (1)	1.5 (4)
Trans violence, lifetime (range=0–4)	0.5 (1.25)	–
Ever arrested or convicted because trans (%)	5.8 (4)	–
Ever denied employment because trans (%)	44.9 (31)	–
Ever harassed by police because trans (%)	27.5 (19)	–
Healthcare provider stigma past six months (range=0–4)	0.4 (0.8)	–

### Stigma and HIV prevention service access among trans MSM

Reported stigma and discrimination were not associated with access to lubricants. Associations between stigma and discrimination variables and HIV testing and condoms are shown in Tables 2 and 3, respectively. Having been arrested or convicted due to being trans was significantly associated with lack of access to condoms; no trans MSM who had been arrested or convicted reported that condoms were completely accessible, as compared to 56.9% of trans MSM without this experience of discrimination. Access to HIV testing was also lower among those who had been arrested or convicted, although this relationship did not reach statistical significance.

**Table 2:** Associations between stigma and access to HIV testing among transgender men (*n*=69) in a global survey of men who have sex with men

Stigma	Testing completely accessible % or *x*¯	Testing not accessible % or *x*¯	*Z* a	*P* b
Violence scale score (median, range=0–4)	0.5	0.75	1.11	0.27
Ever arrested or convicted because trans	0.0%	10.3%	–	0.09
Ever denied employment because trans	43.3%	46.2%	–	0.50
Ever harassed by police because trans	20.0%	33.3%	–	0.17
Healthcare provider stigma, past six months (median, range=0–4)	0.2	0.6	1.94	0.05

a *Z*-test and *P*-value for continuous variables from Wilcoxon rank-sum test

b *P*-value for difference in proportions from Fisher's exact test.

**Table 3:** Associations between stigma and access to condoms among transgender men (*n*=69) in a global survey of men who have sex with men

Stigma	Condoms completely accessible % or *x*¯	Condoms not accessible % or *x*¯	*Z* a	*P* b
Violence scale score (median, range=0–4)	0.5	0.75	0.42	0.68
Ever arrested or convicted because trans	0.0%	12.5%	–	**0.04**
Ever denied employment because trans	48.6%	40.1%	–	0.34
Ever harassed by police because trans	29.7%	25.0%	–	0.43
Healthcare provider stigma, past six months (median, range=0–4)	0.6	0.2	−1.22	0.22

a *Z*-test and *P*-value for continuous variables from Wilcoxon rank-sum test

b *P*-value for difference in proportions from Fisher's exact test. Bolded values indicate statistical significance at *p*<0.05.

In addition, a marginally significant (*p=*0.05) association was found between the frequency of healthcare provider stigma over the past six months and accessibility of HIV testing, with higher provider stigma scores among those for whom HIV testing was not completely accessible. While transphobic violence was not related to any type of service access overall, those who had ever been sexually assaulted due to being trans were less likely to report access to HIV testing, compared to trans MSM who were never sexually assaulted (results not shown; 22.2% vs. 50.0%, *p=*0.04).

### Access to HIV prevention services by transgender status

As shown in Table 4, HIV testing, condoms, and lubricants were completely accessible for 43.5, 53.6, and 26.1% of trans MSM, respectively. In contrast, HIV testing, condoms, and lubricants were completely accessible for 56.9, 54.7, and 39.5% of cisgender MSM, respectively. In conditional logistic regression models, in comparison to age-, region-, and HIV status-matched cisgender controls, trans MSM reported significantly lower odds of perceived access to HIV testing (OR=0.57, 95% CI=0.33, 0.98) and lubricants (OR=0.54, 95% CI=0.30, 0.98). No differences were found with respect to access to condoms. Results of sensitivity analyses with adjustment for SES status were not appreciably different (results not shown).

**Table 4:** Comparing access to HIV prevention services between transgender (*n*=69) and pair-matched cisgender (*n*=276) men^a^ in a global survey of men who have sex with men

	% (*n*)	Odds ratio (95% CL)	*P*
HIV testing completely accessible			**0.04**
Cisgender MSM	56.9 (157)	1.00	
Transgender MSM	43.5 (30)	0.57 (0.33, 0.98)	
Condoms completely accessible			0.87
Cisgender MSM	54.7 (151)	1.00	
Transgender MSM	53.6 (37)	0.96 (0.56, 1.63)	
Condom-compatible lubricants completely accessible			**0.04**
Cisgender MSM	39.5 (109)	1.00	
Transgender MSM	26.1 (18)	0.54 (0.30, 0.98)	

a Matched on age group, region, and HIV status. Bolded values indicate statistical significance at *p*<0.05.

## Discussion

We found partial support for our hypotheses that exposure to anti-transgender violence, discrimination, and healthcare provider stigma would be negatively related to accessibility of HIV prevention service. Access to free or low-cost condoms, and to HIV testing, was lower among those reporting arrest or conviction due to being trans. This may reflect the impact of living in a highly legally-constrained environment, rather than a direct result of arrest or conviction. These findings indicate a need for further research on, and service delivery to address, the sexual health of trans MSM in settings with official and informal legal sanctions against trans people. Ultimately, structural interventions are required to eliminate laws and policies that criminalize trans people and MSM.

Those respondents reporting no or limited access to HIV testing had (marginally significantly) higher past six-month healthcare provider stigma scores. While participants were not asked to evaluate trans cultural competency in rating accessibility of prevention services, it is likely that such considerations would impact perceived accessibility. Past experiences of enacted anti-transgender stigma in healthcare settings, and anticipation of future stigma, are associated with reduced access to healthcare for trans men [22,30]. In addition, providers with stigmatizing attitudes towards gender and sexual minorities may be less aware of available resources for trans patients [19,20], including sexual health services, and less likely to refer patients to them. It is also possible that some reported experiences of provider stigma occurred in the context of sexual health or HIV testing services.

While not statistically significant, among those without complete access to HIV testing, proportions reporting each form of discrimination were consistently higher, as were violence scale scores. This was not the case for condoms or lubricants. We note that with our small trans MSM sample (*n*=69), power to detect significant differences was low. Few data are available regarding utilization of HIV testing services among trans MSM, but there is some evidence to suggest low uptake [31]. Further research could examine the impacts of various manifestations of stigma on access to and utilization of HIV testing among trans MSM, including stigma experienced when accessing testing services and lack of inclusion in testing-related outreach and materials. While the present study only considered trans-specific stigma, trans MSM may additionally face stigma related to their sexual minority identity and/or behaviour. Researchers may wish to consider how gender and sexual stigmas, and intersecting stigmas related to race, ethnicity, class, gender expression, and other aspects of social identity or position interact to impact access for trans MSM [21].

Findings supported our hypothesis that trans MSM would report less access to basic HIV prevention services as compared to cisgender MSM, with the exception that while almost half of trans MSM had inadequate access to condoms, this proportion was quite similar to cisgender MSM. Our findings are consistent with trans men's qualitative reports of barriers to accessing sexual health services, particularly HIV and other sexually transmitted infections testing [10,23]. HIV testing is a pre-requisite for access to HIV treatment and pre-exposure prophylaxis, as well as for prevention of onwards transmission. That trans MSM report less access than their cisgender counterparts (among whom access is already inadequate) indicates that enhancing access to culturally competent testing services should be prioritized. While not explored in this study, the co-provision of transgender healthcare and HIV prevention services may improve service utilization.

In addition to their importance for safer anal intercourse, access to lubricants is crucial for trans MSM on testosterone therapy who engage in receptive vaginal sex. Although not yet studied among trans men, changes to vaginal tissue related to oestrogen deficiency among menopausal cisgender women are well documented, including atrophy, dryness, and loss in elasticity, which increase risk of tissue injury, bleeding, and infection [32]. Trans men report similar symptoms [12] and thus may be at heightened risk of STI or HIV infection related to vaginal intercourse, particularly if they do not have access to condom-compatible lubricants.

One potential explanation for a disparity in access to lubricants but not to condoms relates to the settings in which condom-compatible lubricants are often distributed free of charge, that is, MSM-focused clinical and community venues. Many trans MSM want to receive service in such settings [11], but face perceived and enacted exclusion from them [33]. Similarly, that trans MSM reported less access to HIV testing than their cisgender counterparts may be related to their exclusion from HIV prevention and testing services targeted to MSM. Efforts to understand and reduce in-group stigma that trans MSM face from cisgender MSM and MSM-oriented services may be required to increase access to HIV testing and lubricants.

### Strengths and limitations

The present analysis has several important limitations. As part of a study of MSM, we recruited trans men who align themselves with gay, bisexual, and other MSM communities. The experiences of trans masculine individuals who are attracted to men but do not identify with these terms may differ. Additionally, while specific outreach to trans MSM was conducted, most participants were recruited via predominantly cisgender MSM networks, from which some trans men may be excluded or feel alienated. More broadly, results of this small convenience sample cannot be generalized to trans MSM globally or in any specific setting. Survey participants had email and Internet access and were at least minimally connected to sexual minority communities, and thus, they may have greater access to HIV prevention services than other MSM. Comparisons of service access between trans and cisgender MSM employed matching based on age group, region, and HIV status, but other factors may confound associations between transgender status and access. However, sensitivity analyses indicated that SES (operationalized as ability to meet one's basic needs) did not act as a confounder. Additionally, residual confounding within relatively heterogeneous regional groups is possible. Finally, measures were based on self-report, were not formally validated, and only reflect perceptions of stigma and accessibility; these measures are also subject to recall bias. The two-step method for identifying trans respondents has been evaluated in English, Spanish, and Portuguese [26,34], but its validity across diverse cultures and languages remains to be established [35].

Nevertheless, our study has some notable strengths. It was one of the first studies focused on (cisgender) MSM to include and report on data specific to trans MSM. In addition, representation of trans men was greater than in the two previous reports on trans men in MSM studies (in which trans men constituted 14 of 717 [8] and 32 of 36,063 [26] participants). Strategies for sensitive and valid data collection with trans MSM were developed in collaboration with community members, and outreach was conducted to ensure participation of trans men. Recruitment materials specified trans-inclusivity but were not targeted specifically to trans men. As a result, trans men who are “stealth” (i.e., do not disclose their trans history) may have felt more comfortable participating. The survey was translated into seven languages and open to MSM in any country, and it represents an important step towards reflecting experiences of trans MSM living outside of Canada and the United States. In addition, while need characteristics will impact actual utilization of HIV prevention services, our comparison of perceived service accessibility is unlikely to be confounded by differences in need between cisgender and trans MSM. Utilization of preventative healthcare has been associated with its perceived accessibility, a construct that incorporates considerations of service availability, affordability, physical and geographic accessibility, and cultural competence [35].

## Conclusions

We have provided a first look at access to basic HIV prevention services for trans MSM globally, demonstrating associations with some facets of trans-specific stigma and discrimination (particularly arrest or conviction and healthcare provider stigma), and identifying disparities among MSM based on transgender status. Our study has implications for the conduct and content of future trans and MSM sexual health research, as well as for enhancing HIV prevention service access for trans MSM. Exclusion of trans MSM from research based on their natal sex is arguably unethical and contributes to their invisibility in the HIV response [7,36]. For future MSM-focused survey research, our study highlights the feasibility of including trans men using a two-step method for ascertaining assigned sex at birth and gender identity, and the potential to use the resulting data to examine health disparities—something that cannot be accomplished with trans-specific studies alone. Moreover, in online or interviewer-administered questionnaires, skip patterns can easily be implemented to include questions specific to the experiences of trans respondents. In the future, intentional over-sampling of trans MSM may be required for more complex analyses, such as investigations of potential contributors to disparities in access to HIV services.

Substantively, trans and MSM health research should further explore facilitators and barriers to HIV prevention services for trans MSM and consider implementation and evaluation of interventions to enhance access. In a context of stigma at multiple levels, interventions must also be multi-level and may include legal changes, policy changes in healthcare settings, community-level interventions, and clinical interventions. Examples of potential interventions in these domains include efforts to repeal laws that criminalize gender and sexual minorities, introduction of policies to explicitly include trans MSM in health services targeting MSM, campaigns to raise awareness about HIV prevention services among trans MSM, and integration of HIV testing in trans-friendly primary care.
